# Diet and toenail arsenic concentrations in a New Hampshire population with arsenic-containing water

**DOI:** 10.1186/1475-2891-12-149

**Published:** 2013-11-16

**Authors:** Kathryn L Cottingham, Roxanne Karimi, Joann F Gruber, M Scot Zens, Vicki Sayarath, Carol L Folt, Tracy Punshon, J Steven Morris, Margaret R Karagas

**Affiliations:** 1 Department of Biological Sciences, Dartmouth College, Hanover, NH, USA; 2 School of Marine and Atmospheric Sciences, Stony Brook University, Stony Brook, NY, USA; 3 Department of Epidemiology, Gillings School of Global Public Health at the University of North Carolina, Chapel Hill, NC, USA; 4 Section of Biostatistics and Epidemiology, Geisel School of Medicine at Dartmouth, Hanover, NH, USA

**Keywords:** Biomarkers, Drinking water, Population-based study, Food borne exposure, Rice, Fish

## Abstract

**Background:**

Limited data exist on the contribution of dietary sources of arsenic to an individual’s total exposure, particularly in populations with exposure via drinking water. Here, the association between diet and toenail arsenic concentrations (a long-term biomarker of exposure) was evaluated for individuals with measured household tap water arsenic. Foods known to be high in arsenic, including rice and seafood, were of particular interest.

**Methods:**

Associations between toenail arsenic and consumption of 120 individual diet items were quantified using general linear models that also accounted for household tap water arsenic and potentially confounding factors (e.g., age, caloric intake, sex, smoking) (n = 852). As part of the analysis, we assessed whether associations between log-transformed toenail arsenic and each diet item differed between subjects with household drinking water arsenic concentrations <1 μg/L versus ≥1 μg/L.

**Results:**

As expected, toenail arsenic concentrations increased with household water arsenic concentrations. Among the foods known to be high in arsenic, no clear relationship between toenail arsenic and rice consumption was detected, but there was a positive association with consumption of dark meat fish, a category that includes tuna steaks, mackerel, salmon, sardines, bluefish, and swordfish. Positive associations between toenail arsenic and consumption of white wine, beer, and Brussels sprouts were also observed; these and most other associations were not modified by exposure via water. However, consumption of two foods cooked in water, beans/lentils and cooked oatmeal, was more strongly related to toenail arsenic among those with arsenic-containing drinking water (≥1 μg/L).

**Conclusions:**

This study suggests that diet can be an important contributor to total arsenic exposure in U.S. populations regardless of arsenic concentrations in drinking water. Thus, dietary exposure to arsenic in the US warrants consideration as a potential health risk.

## Background

Exposure to arsenic has been linked to a variety of adverse human health effects, including skin lesions; skin, lung, and bladder cancer; vascular diseases; low birth weight; and potentially diabetes mellitus and increased susceptibility to infection [1-3]. Although human exposure to the more toxic, inorganic forms of arsenic is thought to occur primarily through drinking water [3,4], elevated concentrations of arsenic in certain foods may pose an additional risk to consumers (e.g., [2]). Foods with particularly high total arsenic concentrations include fish and seafood [5-9]; cereals and cereal products, particularly rice and rice-based products [5-7,10,11]; and bran and germ [2]. Using diet data and physiological models to estimate total arsenic exposure, Xue et al. [12] found that fruits and fruit juices, vegetables, and beer and wine also can be important sources of dietary arsenic; more recent studies document high arsenic concentrations in cruciferous vegetables in particular [13]. Importantly, the form of arsenic differs among these different types of food: the arsenic in seafood is primarily in putatively less toxic, organic forms such as arsenobetaine, arsenolipids, and arsenosugars [9,14], while the arsenic in most other items includes both inorganic and organic forms of arsenic that have been associated with toxicity [2].

Estimates of arsenic intake based on dietary patterns and arsenic concentrations in individual food items suggest that food can be a significant source of arsenic in Western diets. For example, a recent, comprehensive report by the European Food Safety Authority (EFSA) concluded that some Europeans, especially children, may be consuming appreciable levels of arsenic through food [2]. Moreover, Xue et al. [12] estimated that the general U.S. population may be at greater risk of exposure to arsenic via food than drinking water, supporting similar conclusions by previous researchers [7,15-17]. Pregnant women, infants, children, the elderly, and those with compromised immune function could be particularly vulnerable to the health effects of dietary arsenic exposure [1,12,18].

Despite these advances, neither the EFSA report [2] nor previous research (e.g., [6]) has specifically evaluated exposure via diet after accounting for exposure to arsenic via the household water supply used for drinking and cooking. Therefore, as part of ongoing studies of arsenic and cancer risk, we evaluated the relationships between long-term diet, as reported by subjects using a validated food-frequency questionnaire, and total arsenic concentrations in toenail clippings in a population with varying exposure to arsenic via household water. Toenail clippings provide a biomarker of exposure over the past 2–18 months [6,19-25]: ingested inorganic arsenic circulating through the body binds irreversibly to the sulfhydryl groups in keratin at the base of the growing nail [22,26], which then grows out to be clipped some months later. Previous research indicates that arsenic in toenail clippings is positively correlated with arsenic concentration in the household water at water arsenic concentrations ≥1 μg/L [19,26-28] but is only weakly associated when concentrations are <1 μg/L [19]. One plausible explanation for the latter is that dietary exposure explains more of the variability in toenail arsenic in individuals with little exposure via drinking water. Here, we evaluate whether associations between overall body burden (as indicated by toenail arsenic concentration) and potential dietary exposure from individual food items differ with exposure via drinking water.

## Methods

### Study population

We analyzed existing data from population-based case–control studies of bladder and skin cancer conducted among 25 to 74 year old residents of New Hampshire [29-33]. Groundwater arsenic concentrations for this population vary from <0.0003 to 180 μg/L [34-36], creating a natural gradient of exposure to inorganic arsenic via drinking water. The Committee for the Protection of Human Subjects (CPHS) of Dartmouth College approved study materials and protocols (current CPHS #10107 & #11697) and participants provided informed consent according to the approved protocol.

### Data collection

In this study, we evaluated the association between arsenic in toenail clippings, household water arsenic, and average daily consumption of 120 different diet items for which data were available. Study participants provided toenail clippings and a household tap water sample for analysis of total arsenic concentration using previously established protocols [19,37]; most of the arsenic in both of these matrices is likely to be in the inorganic form (see e.g., [38] and [22], respectively). Toenail clippings (mass 0.04 ± 0.01 g, mean ± 1 standard deviation [SD]) were analyzed at the University of Missouri Research Reactor Center using standard-comparator instrumental neutron activation analysis (NAA). Nails were washed carefully to remove external contamination, freeze-dried, and then stored in sealed vials until testing [19,37]. Samples, certified reference material, and a keratin quality control sample were irradiated for 60 min at a thermal neutron flux of 8 × 10^13^ neutrons cm^-2^ s^-1^, then live-counted for 2 hours at a sample-to-detector distance of ~10 cm using a high resolution gamma-ray spectrometer after a decay period of ~24 hours. Arsenic was calculated from the 559-keV gamma ray from the decay of As-76, relative to known standards and after correction for physical decay. Quality control samples were within 1 SD of the expected value [19].

Drinking water samples were analyzed in the Dartmouth Trace Element Analysis Core using a Finnigan MAT GmbH ELEMENT high resolution inductively coupled mass spectrometer equipped with an MY hydride generator (Finnigan MAT GmbH, Bremen, Germany) [19]. Samples were acidified to pH 1 with ultrapure nitric acid upon arrival at the laboratory, then spiked with Suprapur H_2_O_2_ (Merck KGaA, Darmstadt, Germany) to 0.01% at least 24 hours prior to analysis. During analysis, hydride generation was used to separate arsenic from ArCl + species, increasing the ability to detect arsenic at concentrations <5 μg/L [19].

Participants also completed a written, validated, semi-quantitative food frequency questionnaire (FFQ) [39,40] to quantify diet over the previous year. An annual FFQ should provide a good match to the time scale over which the toenail clippings provide an integrated measure of exposure [6,37]. The FFQ asked about the consumption of specific portion sizes of 120 different items from seven broad categories (dairy, fruits, vegetables, eggs and meat, breads, beverages, and baked goods) over the previous 12-month period; we analyzed associations between toenail arsenic and each of these diet items. We converted all responses to servings per day using the midpoint of each interval and assuming that a month has 30 days (Additional file 1: Table S1). When participants skipped a question, we set the frequency of consumption to missing. As in MacIntosh et al. [6], we focused on whole foods; the associations between toenail arsenic concentrations and micronutrient and vitamin consumption, both with and without supplements, were analyzed separately [41].

In addition, participants were interviewed, usually in their home, to obtain information on sociodemographic and lifestyle factors (e.g., smoking history, drinking water source, [33]) that may have affected the association between toenail arsenic concentration, drinking water arsenic concentration, and individual diet items.

### Statistical analyses

Prior to analysis, we normalized data on toenail arsenic concentrations using natural-log (ln) transformation. Analyses reported here exclude the 70 subjects who did not report using their household water for drinking and cooking as well as subjects who did not meet the caloric thresholds suggested by Willett [42]: 18 men below 800 calories and 13 above 4000 calories, and three women below 500 calories and four above 3500 calories. We also excluded from the analysis one individual with an extremely high toenail arsenic concentration (7.6 μg/g), which was 420% higher than the next highest concentration [41]. This left us with a sample size of 852 subjects, down from 934.

In all analyses, we accounted for the previously observed non-linear association between toenail arsenic concentrations and household water arsenic concentration [19] by including natural-log transformed household water arsenic, an indicator variable [43] for whether subjects had concentrations of arsenic in household water <1 μg/L or ≥1 μg/L, and their interaction in a general linear model (GLM, SAS version 9.2).

We then evaluated associations with the self-reported, estimated daily rate of consumption of each of the 120 diet items in the FFQ. In the first stage of the analysis, we determined whether the association between water-corrected toenail arsenic concentration and each diet item differed between the two household water arsenic groups (<1 μg/L vs. ≥1 μg/L) using an interaction between the consumption rate of the diet item and the indicator variable for household water arsenic. If there was no statistically significant (α = 0.05) interaction between the diet item and the water exposure group, we concluded that the association between water-corrected toenail arsenic and the diet item was not affected by water exposure, and fit a GLM to the full dataset in the second stage of analysis (Model 1). However, if there was a statistically significant interaction between the diet item and the water exposure group, we concluded that the association between water-corrected toenail arsenic and the diet item differed between the two water exposure groups and so conducted analyses separately for the two groups (Models 2a and 2b). The slope coefficients ($\hat{\beta }$) for each dietary item have the units natural-log transformed (toenail arsenic concentrations, μg/g) · (servings/d)^-1^.

For those diet items for which the slope coefficient was statistically significant for the appropriate model (1 or 2a/2b), we evaluated robustness to extreme values in the predictors by looking at unadjusted scatterplots, then recalculating regression coefficients after systematically deleting visually apparent outliers. Seven diet items were no longer significant after removal of such values and were not considered further.

Although associations between toenail arsenic and demographic characteristics such as age have previously been described [19], the mechanisms behind these associations have not been elucidated. For example, we do not know whether age directly affects toenail arsenic, or whether age influences diet, which in turn influences exposure as indicated by toenail concentrations. We therefore reported “crude” unadjusted associations between water-corrected toenail arsenic and each diet item, as well as analyses after adjustment for covariates that were deemed important from previous literature [19,44,45], biological plausibility, and univariate associations [41]. We adjusted for four categorical variables (sex, smoking status [never/ever], season of toenail collection, case–control status [control, bladder cancer, basal cell carcinoma, squamous cell carcinoma]) and three continuous variables (age, daily intake of water from the household water source [ounces · d^-1^], and total energy intake [kcal · d^-1^]).

To help interpret regression coefficients from these adjusted models, we determined the percent change in predicted (back-transformed) toenail arsenic concentrations between 5^th^ percentile and 95^th^ percentile consumers for each food, using an approach similar to that described in Gruber et al. [41]. Predictions were made for non-smoking, control subjects whose toenails were collected during the most common season (fall), separately for males and females at the mean age, caloric consumption, and water consumption for their sex. For Model 1 foods (those for which associations were consistent across household water arsenic concentrations), we used the overall median household water concentration. For Model 2 foods (those for which associations differed between water arsenic exposure groups), we used the median household water concentration for the appropriate exposure group.

We accounted for multiple testing across the individual foods using the false discovery rate (FDR) procedure implemented in the R package qvalue [46]. Specifically, we calculated the Q-value, the minimum FDR at which a test may be called statistically significant [47], from the combined list of P-values for the association with each of the 120 foods, as generated by the appropriate model (1, 2a or 2b). We considered variables with a Q-value > 0.1 as less likely due to multiple testing.

## Results

The 852 individuals in our study had a median arsenic concentration in the household tap water of 0.30 μg/L and a median toenail arsenic concentration of 0.085 μg/g. Mean arsenic concentrations in toenails were lower for those with household water arsenic concentrations <1 μg/L than in the group with household water arsenic ≥1 μg/L (Table 1). Fifty-two individuals had household water arsenic concentrations at or above the EPA drinking water standard of 10 μg/L.

**Table 1:** Summary statistics for the study population

**Variable**	**Total**	**Water arsenic**	**Water arsenic**
	**population**	**<1 μg/L**	**≥1 μg/L**
Drinking water arsenic (μg/L)	2.72 (0.35)	0.27 (0.01)	10.86 (1.36)
Toenail arsenic (μg/g)	0.12 (0.005)	0.10 (0.004)	0.19 (0.02)
Age (years)	61.1 (0.3)	61.2 (0.4)	60.7 (0.7)
Intake from household tap water (# 8 oz. glasses/d)	5.0 (0.1)	5.1 (0.1)	5.0 (0.2)
Energy intake (kcal/d)	1920 (22)	1912 (25)	1944 (42)
Sex			
Female	330 [61]	254 [39]	76 [39]
Male	522 [39]	401 [61]	120 [61]
Smoking Status			
Never Smoked	275 [32]	208 [32]	67 [34]
Has Smoked	577 [68]	447 [68]	130 [66]
Season			
Winter	168 [20]	127 [19]	41 [21]
Spring	171 [20]	137 [21]	34 [17]
Summer	246 [29]	175 [27]	71 [36]
Fall	267 [31]	216 [33]	51 [26]
Case–control Status			
Control	211 [25]	167 [25]	44 [22]
Bladder Cancer	248 [29]	168 [28]	62 [31]
Basal Cell Skin Cancer	198 [23]	150 [23]	48 [24]
Squamous Cell Skin Cancer	195 [23]	152 [23]	43 [22]

*Legend*: Summary statistics are provided first for the total study population (n = 852), and then for the two household drinking water exposure groups: <1 μg/L (n = 655) and ≥1 μg/L (n = 197). Continuous variables are given as mean (SE) and categorical variables as N [%].

Individuals with <1 μg/L vs. ≥1 μg/L water arsenic were similar with respect to gender, smoking status, case status, and the season of toenail collection (Table 1). Further, there was comparability between groups in age, energy intake, and amount of water consumed from the household source each day (Table 1).

In interpreting the results of our general linear models, we focused on the foods known to be high in arsenic (e.g., rice, seafood [2]) and those foods that were statistically significant after correction for multiple testing (Q-values ≤0.1), based on the models that included potential confounders. Although the statistical significance of some relationships depended on whether we used crude or adjusted models to evaluate associations between water-corrected toenail arsenic and diet, the direction of effects (i.e., whether associations were positive or negative) was robust to the inclusion of potential confounding variables.

For 116 of the 120 foods investigated, the association between water-corrected ln-transformed toenail arsenic and diet did not differ by whether arsenic was present in the drinking water supply at concentrations ≥1 μg/L (i.e., the interaction term between the indicator variable and the diet item was not statistically significant). We found a positive association with dark meat fish, a category that includes tuna steaks, mackerel, salmon, sardines, bluefish, and swordfish and which accounted for about 1.5% of the variation in ln-transformed toenail arsenic in our adjusted model. Based on this model, toenail arsenic is predicted to be 7.4% higher among both males and females eating these fish once weekly (95^th^ percentile consumers) as compared to less than once per month (5^th^ percentile consumers, Table 2). In contrast, we did not detect a clear relationship between toenail arsenic and rice consumption (adjusted model $\hat{\beta }$ for brown rice = 0.23 ± 0.22 (1 SE), *P* = 0.29; $\hat{\beta }$ for white rice = 0.065 ± 0.15, *P* = 0.67), although toenail arsenic was positively associated with two other types of grains: bran and a miscellaneous category that included bulgur, kasha, couscous, and other grains (Table 2).

**Table 2:** Diet items that were associated with toenail arsenic across the whole population

		**Crude models**^**1**^			**Adjusted models**^**2**^				
								**% change from 5**^**th **^**to 95**^**th **^**percentile consumers**^**3**^	
**Category**	**Item**	$\hat{\beta }$ **± SE**^**4**^	***P***	**partial R**^**2**^	$\hat{\beta }$ **± SE**	***P***	**partial R**^**2**^	**Males**	**Females**
Meats	Dark meat fish (tuna steak, mackerel, salmon, sardines, bluefish, swordfish, 3–5 oz)	0.50 ± 0.22	0.025	0.8%	0.62 ± 0.22	0.004	1.5%	7.4	7.4
	Beef, calf, or pork liver (3–4 oz.)	-1.21 ± 0.52	0.019	0.9%	-0.89 ± 0.50	0.073	1.1%	-5.8	-5.8
	Hamburger (1 patty)	-0.32 ± 0.15	0.034	-0.1%	-0.32 ± 0.15	0.038	-0.3%	-12.6	-12.6
Dairy	Eggs (1)	-0.12 ± 0.05	0.036	0.4%	-0.10 ± 0.05	0.060	0.3%	-7.7	-7.7
Grains	Bran, added to food (1 Tbsp)	0.14 ± 0.08	0.094	0.4%	0.15 ± 0.08	0.048	1.0%	0.9	6.0
	Other grains (bulgur, kasha, couscous, etc.: 1 cup)	1.26 ± 0.46	0.006	1.9%	0.80 ± 0.45	0.074	2.4%	5.5	12.1
	Cold breakfast cereal (1 cup)	-0.12 ± 0.05	0.014	1.1%	-0.10 ± 0.05	0.039	1.2%	-11.6	-11.6
Fruits	Cantaloupe (1/4 melon)	0.22 ± 0.12	0.079	0.7%	0.25 ± 0.12	0.039	1.0%	9.7	9.7
Vegetables	**Brussels sprouts (1/2 cup)**	**0.69** ± **0.27**	**0.010**	**0.3**%	**0.89** ± **0.26**	**0.001**	**1.0**%	**10.4**	**10.4**
	Raw carrots (1/2 carrot or 2–4 sticks)	0.18 ± 0.08	0.025	0.4%	0.15 ± 0.08	0.048	0.7%	7.9	14.9
	Celery (4" stick)	0.25 ± 0.10	0.010	0.3%	0.29 ± 0.09	0.002	0.8%	11.3	21.8
	Eggplant or zucchini (1/2 cup)	0.44 ± 0.18	0.017	0.8%	0.37 ± 0.18	0.047	1.2%	6.5	20.8
	Tofu or soybeans (3–4 oz.)	0.58 ± 0.26	0.025	0.3%	0.46 ± 0.25	0.063	0.7%	3.1	6.8
	Salsa/red chili sauce (1 Tbsp)	0.53 ± 0.18	0.003	0.6%	0.33 ± 0.17	0.057	0.9%	4.8	4.8
Beverages	**Beer, regular (1 glass, bottle, or can)**	**0.11** ± **0.03**	**0.001**	**0.8**%	**0.11** ± **0.03**	**0.001**	**1.1**%	**32.0**	**4.9**
	Red wine (5 oz glass)	0.12 ± 0.05	0.012	0.5%	**0.14 ± 0.05**	0.003	1.3%	12.9	5.3
	**White wine (5 oz glass)**	**0.27** ± **0.06**	**0.000**	**1.9**%	**0.27** ± **0.06**	**0.000**	**2.4**%	**12.1**	**23.3**

*Legend:* Model results for the diet items with statistically significant associations with ln-transformed toenail arsenic, adjusted for tap water arsenic. Sample sizes ranged from n = 807-843. Bolding indicates a Q-value < 0.1.

^1^“Crude” indicates the model with adjustment for tap water arsenic only.

^2^“Adjusted” indicates the model with adjustment for tap water arsenic, age, sex, caloric intake, daily water consumption, smoking status, case–control status, and season of toenail collection.

^3^These columns summarize the percent change in predicted toenail arsenic concentration from 5^th^ to 95^th^ percentile consumers for males and for females; gender is included since males and females consumed some diet items (e.g., beer, wine) differently.

^4^Units for the estimated coefficients are natural-log transformed (toenail arsenic concentration, μg/g) (servings/d)^-1^.

Of all the diet items evaluated, only beer, white wine, and Brussels spouts were statistically significant after correction for multiple testing (Table 2). Alcoholic beverages – beer and white wine, and to a lesser extent red wine – were positively associated with toenail arsenic; partial R^2^ for these effects ranged from 1.1-2.4% in the adjusted models. Associations with alcohol consumption differed by sex. In men, predicted toenail arsenic concentrations are >30% higher in those consuming 2.5 beers/day (95^th^ percentile consumers) as compared to non-consumers (5^th^ percentile consumers). In women, predicted toenail arsenic is >20% higher for those drinking 5–6 glasses of white wine per week (95^th^ percentile consumers) compared to those who did not drink this beverage (5^th^ percentile consumers). Increased consumption of Brussels sprouts was also positively related to toenail arsenic: the models predict a 10% increase in toenail arsenic when consumption increases from never eating Brussels sprouts (5^th^ percentile consumers) to eating them once per week (95^th^ percentile consumers) (Table 2).

Other associations between diet items and toenail arsenic were not statistically significant after correction for multiple testing (Table 2). For example, we detected positive associations with tofu and other soy products, cantaloupe, raw carrots, celery, eggplant or zucchini, and red chili sauce, and inverse associations with consumption of cold breakfast cereals; eggs; beef, calf, or pork liver; and hamburger (Table 2).

The associations for four foods differed according to household tap water arsenic concentration (Table 3). For cooked oatmeal/oat bran and beans or lentils, the association was stronger among those with ≥1 μg/L water arsenic. For hot dogs and liquor, the direction of the association changed for those with <1 μg/L vs. ≥1 μg/L water arsenic (Table 3). None of these associations remained statistically significant after adjustment for multiple testing.

**Table 3:** Diet items that were differentially associated with toenail arsenic depending on household water arsenic

		**Tap water arsenic <1 μg/L**					**Tap water arsenic** ≥ **1 μg/L**				
**Category**	**Food**	$\hat{\beta }$ ± **SE **^**2**^	***P***	**partial R**^**2**^	**%Change from 5**^**th **^**to 95**^**th **^**percentile consumers**^**1**^		$\hat{\beta }$ ± **SE**	***P***	**partial R**^**2**^	**% Change from 5**^**th **^**to 95**^**th **^**percentile consumers**^**1**^	
					**Males**	**Females**				**Males**	**Females**
Meats	Hot dogs (beef or pork)	-0.457 ± 0.192	0.018	0.8%	-18.2	-6.5	0.564 ± 0.356	0.115	1.1%	34.7	10.5
Grains	Cooked oatmeal or cooked oat bran (1 cup)	0.085 ± 0.099	0.394	0.8%	1.4	2.6	0.539 ± 0.178	0.003	3.2%	18.2	35.8
Vegetables	Beans or lentils, baked, dried, or soup (1/2 cup)	0.194 ± 0.201	0.335	0.6%	6.6	6.6	1.144 ± 0.398	0.005	2.6%	30.0	30.0
Beverages	Liquor, e.g., whiskey, gin, vodka (1 drink or shot)	0.080 ± 0.034	0.019	1.5%	11.7	4.5	-0.119 ± 0.078	0.126	-0.3%	-16.3	-6.9

*Legend:* Model results for the diet items for which the associations with ln-transformed toenail arsenic concentration, adjusted for tap water arsenic concentrations and potential confounders, depended on whether the household tap water arsenic concentration was above or below 1 μg/L. Sample sizes ranged from n = 627-648 for tap water arsenic <1 μg/L and n = 194-195 for tap water arsenic ≥ 1 μg/L.

^1^These columns summarize the percent change in predicted toenail arsenic concentration from 5^th^ to 95^th^ percentile consumers for males and for females; gender is included since males and females consumed some diet items (e.g., beer, wine) differently.

## Discussion

We found that increased consumption of a number of individual diet items, including some but not all of the items expected to be high in arsenic concentrations, was associated with increasing toenail arsenic concentrations in this U.S. population. Importantly, although toenail arsenic increased with household water arsenic, we detected few interactions between household water arsenic and diet; this suggests that responses to dietary arsenic exposure were not highly sensitive to exposure via water.

### Grains, especially rice

We expected to find an association between toenail arsenic and rice consumption because elevated concentrations of arsenic in rice are well documented, both in the U.S. [48-50] and in Southeast Asia [51-54]. Moreover, previous studies have found positive associations between rice consumption and arsenic concentrations in both urine [55-58] and toenails [6]. However, rice consumption was relatively low in this study population: the median study participant reported eating no brown rice and eating white rice just 1–3 times per month. By contrast, consumption rates were higher in studies relating rice consumption and urinary arsenic concentrations [55-58], consistent with a *per capita* rice consumption in the United States of about 0.4 cup of cooked rice per day (derived from USDA commodity consumption data [59]), with some sub-populations consuming up to 2.2 cups per day [60]. Thus, consumption of rice grains in this study population was probably not sufficiently high to leave a signal in a long-term biomarker like toenail clippings. More work is needed to evaluate the association between rice consumption and long-term biomarkers like toenail clippings in a population that regularly consumes rice.

### Fish and seafood

Many previous studies have found that fish have high total arsenic concentrations compared to other food items [5,7] and that fish and seafood contribute a large part of human exposure to total arsenic [2,5-7,12,61]. However, the arsenic in these items is expected to be predominantly in organic forms that are excreted from the body without undergoing biotransformation, such as arsenobetaine and arsenocholine [9]. Thus, our finding of elevated toenail arsenic – which is primarily in inorganic forms [22] – in subjects who consumed more dark meat fish (tuna steak, mackerel, salmon, sardines, bluefish, or swordfish), but not more fish overall, is somewhat unexpected. We do not have data on arsenic speciation for the fish consumed by our study population, but speculate that the forms of arsenic in these types of fish may be qualitatively different from other types of seafood: that is, some of the organic arsenic in these fish might be biotransformed to inorganic forms within the body that then circulate through the bloodstream before being incorporated into nails. This speculation will need to be assessed in further work that includes detailed data on the species of arsenic found in an individual’s diet as well as both urinary and toenail arsenic biomarkers.

Negative associations with toenail arsenic were found for several foods that may be considered alternatives to fish: eggs; beef, calf, or pork liver; and hamburger in the population as a whole, and hot dogs in the group with drinking water arsenic <1 μg/L. MacIntosh et al. [6] suggested that individuals who tend to consume these types of foods tend not to eat as much seafood. Future analyses exploring dietary patterns of “fish eaters” and “non-fish eaters” in other populations may help to further elucidate those at highest risk of arsenic exposure via diet.

#### Alcoholic beverages

Our findings of increased toenail arsenic with increased consumption of beer and wine are consistent with previous modeling studies [12] as well as epidemiological studies with both toenail [6] and urinary [62-64] arsenic biomarkers. This study was not designed to address the mechanisms behind this finding, but we can speculate based on previous research that high arsenic content in these beverages and/or impairment of arsenic detoxification processes within the body may be responsible. For example, beer and wine may themselves be a source of dietary arsenic due to contamination of key ingredients such as hops, rice, and grapes [3,38,65,66]. Alternatively, the use of diatomaceous earth in filtering these beverages prior to consumption could be responsible for such an association [67]. Moreover, past or current alcohol consumption was associated with increased total urinary arsenic concentrations in a study of bladder cancer in Taiwan [62]. Additionally, among individuals exposed to varying levels of water arsenic contamination, consumers of one or more alcoholic beverages per week had significantly higher proportions of inorganic urinary arsenic species when compared to individuals who consumed no alcoholic beverages [63,64], suggesting that alcohol may impair the body’s ability to metabolize inorganic arsenic. Unfortunately, we could not address the issue of alcohol effects on arsenic metabolism as nearly all toenail arsenic is in the inorganic form [22], and few data are available on arsenic speciation in these beverages. Nonetheless, our results confirm previous studies suggesting that alcoholic beverages should be taken into account when evaluating exposure to arsenic via diet [12].

### Fruits and vegetables

Although we did not observe any associations with particular fruits or vegetables containing specific vitamins or micronutrients known to enhance arsenic detoxification [41,45], we did find that several vegetables and one fruit were positively associated with water-corrected toenail arsenic, consistent with the modeling study of Xue et al. [12]. However, only the association with Brussels sprouts remained statistically significant after correction for multiple testing. This finding is consistent with recent studies documenting high concentrations of arsenic in Brussels sprouts and other cruciferous vegetables [13] that may result from their high concentrations of sulfur; arsenite is known to bind preferentially to sulfur-containing compounds [68] as part of cellular detoxification of arsenic in plants [69]. As such, further evaluation of Brussels sprouts and other cruciferous plants containing high concentrations of sulfur [70] may be warranted, especially in geographic areas where soils or irrigation water may contain high concentrations of arsenic.

### Foods cooked in water

In addition to Brussels sprouts, toenail arsenic was related to consumption of several other foods that are often – but not always – cooked in water, including oatmeal and legumes (beans or lentils). This was more evident for the sub-population with household water arsenic concentrations ≥1 μg/L, although not statistically significant after correction for multiple comparisons. These relationships might reflect arsenic exposure from cooking water rather than from the uncooked foods themselves. However, we do not have information about the exact processes used in preparing these foods, and there were no associations for many other foods cooked in water (e.g., rice, pasta, potatoes, other vegetables). Thus, it is not clear whether it is the water or these foods themselves that are driving this association.

The EFSA report [2] noted that cooking foods in contaminated water could increase dietary arsenic exposure. Arsenic concentrations in cooking water, the type of food processing, time, temperature, and cooking medium can all affect arsenic concentrations in prepared final products. For example, arsenic concentrations in prepared rice [71-73] and vegetables [74] can be higher than in the raw foods when cooked in arsenic-contaminated water. However, cooking rice in excess water – even if the water itself is contaminated with arsenic – can reduce the arsenic concentrations in the prepared product [75]. Why Brussels sprouts, oatmeal and beans/lentils emerged from our analysis, but not other foods that are often cooked in water, such as rice, requires further scrutiny and replication in other study populations.

### Potential limitations

There are some limitations to this study. First, as noted above, we do not have information about the species of arsenic – inorganic versus organic, or the kinds of organic arsenic – consumed by the study participants. Thus, although we can report associations between individual diet items and toenail arsenic, which reflects inorganic arsenic circulating in the body [22,25], we cannot infer the potential toxicity of arsenic exposure via diet. Second, although toenails can have limitations as biomarkers due to variability in growth rate among individuals, the risk of external contamination, and inconsistent protocols for collection and analysis [23], we have minimized these problems in this study by comparing toenail samples to diet information over a 12 month time frame, collecting toenails immediately after bathing and sonicating them prior to analysis, and using standardized analytical procedures for all subjects [41]. Third, although we have matched the temporal scale of our dietary information to that represented by toenail clippings, food frequency questionnaires are less precise relative to other tools such as dietary records [76]. Because our data on consumption rates are likely to be less precise than our estimates of toenail arsenic concentration, we have violated the assumption that the predictor variables are known more precisely than the response variable. Fortunately, this violation tends to bias results towards the null [77], and so should not result in false positive findings. Finally, while we adjusted for case status, many of the participants were cancer cases. Although this could affect the generalizability of our findings since it is plausible that these individuals process arsenic differently than non-cancer cases [41], the associations with dark meat fish, white wine, beer and Brussels sprouts were also present in analyses only of control subjects (data not shown).

## Conclusions

We found that arsenic concentrations in toenail clippings, a known biomarker of exposure [19,21,23], were correlated with individual diet items, particularly alcoholic beverages and Brussels sprouts, but also dark meat fish. Some foods cooked in water also were associated with higher toenail arsenic concentrations, especially among those with higher water arsenic concentrations, suggesting that exposure might be reduced by using alternative water sources in cooking or by using alternative cooking procedures. The fact that several diet items each accounted for 1-2% of the variability in log-transformed toenail arsenic even after correction for water exposure suggests that food is an exposure route likely to impact the population as a whole, regardless of drinking water arsenic concentrations. Further research is needed to identify patterns of dietary exposure that may pose particular risk, especially in populations expected to be vulnerable to exposure, such as pregnant women and infants [2].

## Competing interests

The authors declare no competing financial interests with this work.

## Authors’ contributions

KLC, RK, MRK, CLF and MSZ designed the research and conducted and interpreted analyses. KLC, RK and MRK drafted the manuscript. MRK conceived and designed the parent study, obtained funding, and collected data. JFG and TP provided interpretations of results. VS coordinated data collection and provided technical support, and JSM collected data. All authors critically revised the manuscript and have read and approved the manuscript.

## Supplementary Material

Additional file 1: Table S1 Conversions from FFQ responses to servings per day, assuming a 30-day month. Click here for file
