# Supplementary material for: Diet and toenail arsenic concentrations in a New Hampshire population with arsenic-containing water
Source: Nutr J. 2013 Nov 16;12:149. doi: 10.1186/1475-2891-12-149 (PMC3907042; doi:10.1186/1475-2891-12-149)
Supplement: Additional file 1: Table S1 — Conversions from FFQ responses to servings per day, assuming a 30-day month. [file 1475-2891-12-149-S1.pdf]

Additional Table 1. Conversions from FFQ responses to servings per day, assuming a 30-day month.

**Response Option**	**Midpoint of Interval**	**Servings per Day**
Never or less than once a month	0	0
1-3 times per month	2 times/month	0.0667
Once per week	1 time/week	0.1429
2-4 times per week	3 times/week	0.4286
5-6 times per week	5.5 times/week	0.7857
Once per day	1 time/day	1
2-3 times per day	2.5 times/day	2.5
4-5 times per day	4.5 times/day	4.5
6+ times per day	6 times/day	6
